# Tetra­chloridobis(dibenzyl sulfoxide-κ*O*)tin(IV)

**DOI:** 10.1107/S1600536811015704

**Published:** 2011-05-07

**Authors:** Thy Chun Keng, Kong Mun Lo, Seik Weng Ng

**Affiliations:** a Department of Chemistry, University of Malaya, 50603 Kuala Lumpur, Malaysia

## Abstract

The six-coordinate Sn^IV^ atom in the title compound, [SnCl_4_(C_14_H_14_OS)_2_], exists in a *cis*-SnCl_4_O_2_ octa­hedral geometry. Of the four Cl atoms, two are close to adjacent S atoms [Cl⋯S = 3.320 (1) and 3.376 (1) Å]; the Sn—Cl bonds involving these two Cl atoms are longer than the other two Sn—Cl bonds.

## Related literature

For the SnCl_4_(DMSO)_2_ adduct (DMSO is dimethyl sulfoxide), see: Kisenyi *et al.* (1985 ▶). For the tetra­hydro­thio­phene-1-oxide adduct, see: Howie *et al.* (2010 ▶).
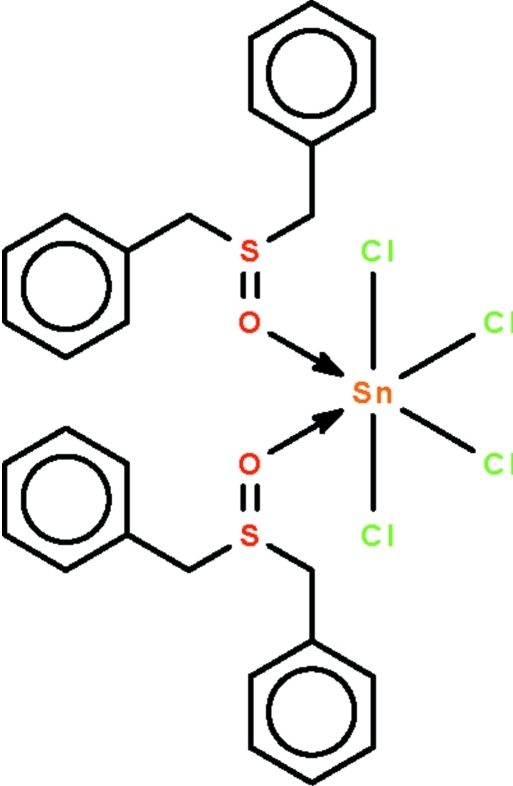


## Experimental

### 

#### Crystal data

[SnCl_4_(C_14_H_14_OS)_2_]
*M* *_r_* = 721.11
Triclinic, 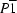

*a* = 10.7982 (1) Å
*b* = 11.1469 (1) Å
*c* = 14.9456 (2) Å
α = 80.8623 (6)°
β = 87.8310 (5)°
γ = 61.4279 (6)°
*V* = 1558.31 (3) Å^3^
*Z* = 2
Mo *K*α radiation
μ = 1.32 mm^−1^
*T* = 100 K
0.30 × 0.30 × 0.30 mm

#### Data collection

Bruker SMART APEX diffractometer
Absorption correction: multi-scan (*SADABS*; Sheldrick, 1996 ▶) *T*_min_ = 0.693, *T*_max_ = 0.693
14537 measured reflections
7139 independent reflections
6609 reflections with *I* > 2σ(*I*)
*R*_int_ = 0.019

#### Refinement

*R*[*F* ^2^ > 2σ(*F* ^2^)] = 0.028
*wR*(*F* ^2^) = 0.073
*S* = 1.05
7139 reflections
334 parameters
H-atom parameters constrained
Δρ_max_ = 0.83 e Å^−3^
Δρ_min_ = −1.02 e Å^−3^

### 

Data collection: *APEX2* (Bruker, 2009 ▶); cell refinement: *SAINT* (Bruker, 2009 ▶); data reduction: *SAINT*; program(s) used to solve structure: *SHELXS97* (Sheldrick, 2008 ▶); program(s) used to refine structure: *SHELXL97* (Sheldrick, 2008 ▶); molecular graphics: *X-SEED* (Barbour, 2001 ▶); software used to prepare material for publication: *publCIF* (Westrip, 2010 ▶).

## Supplementary Material

Crystal structure: contains datablocks global, I. DOI: 10.1107/S1600536811015704/bt5525sup1.cif

Structure factors: contains datablocks I. DOI: 10.1107/S1600536811015704/bt5525Isup2.hkl

Additional supplementary materials:  crystallographic information; 3D view; checkCIF report

## Figures and Tables

**Fig. 1.**
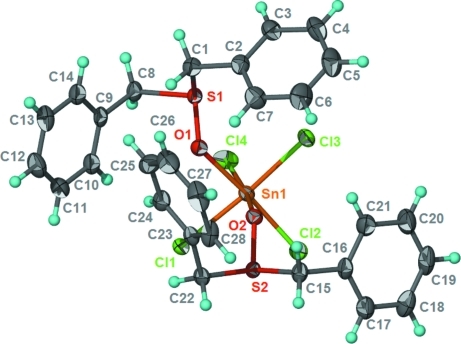
Anisotropic displacement ellipsoid plot (Barbour, 2001) of SnCl4(C14H14OS)2 at the 70% probability level; hydrogen atoms are drawn as spheres of arbitrary radius.

## supplementary crystallographic information

### Comment

The attempted synthesis of the dibenzyl sulfoxide adduct of dibenzyltin chloride resulted in the cleavage both tin-carbon bonds to yield the 1:2 stannic chloride–dibenzyl sulfoxide adduct (Scheme I, Fig. 1). The six-coordinate Sn^IV^ atom exists in *cis*-SnCl_4_O_2_ octahedral geometry (Fig. 1). The adduct exists as a discrete molecule and there are no chlorine···chlorine contacts. The corresponding DMSO adduct shows a similar geometry (Kisenyi *et al.*, 1985) as does the tetrahydrothiophenene-1-oxide adduct (Howie *et al.*, 2010).

### Experimental

Dibenzyltin dichloride (0.37 g, 1 mmol) and dibenzylsulfoxide (0.46 g, 2 mmol) were heated in ethanol (100 ml) for an hour. The solution was filtered and then set aside for the growth of colorless crystals.

### Refinement

H-atoms were placed in calculated positions (C—H 0.95 to 0.99 Å) and were included in the refinement in the riding model approximation, with *U*(H) set to 1.2 times *U*_eq_(C). In the final difference Fourier map, the large peak is in the vicinity of Sn1 and the deepest hole is in the vicinity of the same atom.

### Crystal data

**Table:**

[SnCl_4_(C_14_H_14_OS)_2_]	*Z* = 2
*M**_r_* = 721.11	*F*(000) = 724
Triclinic, *P*1	*D*_x_ = 1.537 Mg m^−^^3^
Hall symbol: -P 1	Mo *K*α radiation, λ = 0.71073 Å
*a* = 10.7982 (1) Å	Cell parameters from 9933 reflections
*b* = 11.1469 (1) Å	θ = 2.3–28.3°
*c* = 14.9456 (2) Å	µ = 1.32 mm^−^^1^
α = 80.8623 (6)°	*T* = 100 K
β = 87.8310 (5)°	Cuboic, colorless
γ = 61.4279 (6)°	0.30 × 0.30 × 0.30 mm
*V* = 1558.31 (3) Å^3^	

### Data collection

**Table:**

Bruker SMART APEX diffractometer	7139 independent reflections
Radiation source: fine-focus sealed tube	6609 reflections with *I* > 2σ(*I*)
graphite	*R*_int_ = 0.019
ω scans	θ_max_ = 27.5°, θ_min_ = 1.4°
Absorption correction: multi-scan (*SADABS*; Sheldrick, 1996)	*h* = −14→14
*T*_min_ = 0.693, *T*_max_ = 0.693	*k* = −14→14
14537 measured reflections	*l* = −19→19

### Refinement

**Table:**

Refinement on *F*^2^	Primary atom site location: structure-invariant direct methods
Least-squares matrix: full	Secondary atom site location: difference Fourier map
*R*[*F*^2^ > 2σ(*F*^2^)] = 0.028	Hydrogen site location: inferred from neighbouring sites
*wR*(*F*^2^) = 0.073	H-atom parameters constrained
*S* = 1.05	*w* = 1/[σ^2^(*F*_o_^2^) + (0.0353*P*)^2^ + 1.7363*P*] where *P* = (*F*_o_^2^ + 2*F*_c_^2^)/3
7139 reflections	(Δ/σ)_max_ = 0.001
334 parameters	Δρ_max_ = 0.83 e Å^−^^3^
0 restraints	Δρ_min_ = −1.02 e Å^−^^3^

### Fractional atomic coordinates and isotropic or equivalent isotropic displacement parameters (Å^2^)

**Table:**

	*x*	*y*	*z*	*U*_iso_*/*U*_eq_	
Sn1	0.596648 (15)	0.444875 (15)	0.252840 (10)	0.01653 (5)	
Cl1	0.38718 (6)	0.50368 (6)	0.34078 (4)	0.02255 (12)	
Cl2	0.73128 (6)	0.23980 (6)	0.35805 (4)	0.02240 (12)	
Cl3	0.79172 (6)	0.41742 (6)	0.16256 (4)	0.02173 (11)	
Cl4	0.51422 (7)	0.34402 (7)	0.15481 (4)	0.02784 (13)	
S1	0.46614 (6)	0.65868 (6)	0.06715 (4)	0.01692 (11)	
S2	0.63003 (5)	0.55337 (5)	0.43151 (4)	0.01626 (11)	
O1	0.47446 (16)	0.64050 (16)	0.17213 (10)	0.0186 (3)	
O2	0.63931 (16)	0.56674 (16)	0.32657 (10)	0.0180 (3)	
C1	0.4837 (2)	0.8131 (2)	0.03527 (16)	0.0220 (5)	
H1A	0.4505	0.8534	−0.0287	0.026*	
H1B	0.4254	0.8828	0.0739	0.026*	
C2	0.6361 (2)	0.7760 (2)	0.04695 (17)	0.0230 (5)	
C3	0.7306 (3)	0.7160 (3)	−0.01962 (17)	0.0261 (5)	
H3	0.6970	0.7040	−0.0736	0.031*	
C4	0.8721 (3)	0.6743 (3)	−0.0072 (2)	0.0336 (6)	
H4	0.9359	0.6332	−0.0524	0.040*	
C5	0.9213 (3)	0.6923 (3)	0.0710 (2)	0.0390 (7)	
H5	1.0188	0.6634	0.0795	0.047*	
C6	0.8287 (3)	0.7522 (3)	0.1369 (2)	0.0375 (6)	
H6	0.8627	0.7655	0.1903	0.045*	
C7	0.6863 (3)	0.7931 (3)	0.12542 (19)	0.0294 (5)	
H7	0.6232	0.8328	0.1713	0.035*	
C8	0.2791 (2)	0.7253 (3)	0.04071 (16)	0.0219 (5)	
H8A	0.2587	0.7669	−0.0242	0.026*	
H8B	0.2580	0.6471	0.0502	0.026*	
C9	0.1836 (2)	0.8315 (2)	0.09659 (16)	0.0211 (5)	
C10	0.1443 (2)	0.7906 (3)	0.18065 (17)	0.0250 (5)	
H10	0.1788	0.6949	0.2027	0.030*	
C11	0.0553 (3)	0.8881 (3)	0.23263 (18)	0.0307 (6)	
H11	0.0295	0.8589	0.2902	0.037*	
C12	0.0036 (3)	1.0280 (3)	0.2011 (2)	0.0334 (6)	
H12	−0.0575	1.0948	0.2367	0.040*	
C13	0.0418 (3)	1.0697 (3)	0.1173 (2)	0.0305 (6)	
H13	0.0064	1.1655	0.0954	0.037*	
C14	0.1313 (3)	0.9726 (3)	0.06500 (18)	0.0258 (5)	
H14	0.1572	1.0022	0.0076	0.031*	
C15	0.7840 (2)	0.5654 (2)	0.46182 (16)	0.0207 (4)	
H15A	0.7877	0.6433	0.4219	0.025*	
H15B	0.7804	0.5812	0.5254	0.025*	
C16	0.9120 (2)	0.4305 (3)	0.45052 (17)	0.0224 (5)	
C17	0.9583 (3)	0.3185 (3)	0.52021 (19)	0.0289 (5)	
H17	0.9121	0.3281	0.5760	0.035*	
C18	1.0721 (3)	0.1923 (3)	0.5085 (2)	0.0358 (6)	
H18	1.1032	0.1156	0.5562	0.043*	
C19	1.1403 (3)	0.1780 (3)	0.4276 (2)	0.0377 (7)	
H19	1.2188	0.0918	0.4200	0.045*	
C20	1.0945 (3)	0.2887 (3)	0.3583 (2)	0.0397 (7)	
H20	1.1411	0.2786	0.3026	0.048*	
C21	0.9800 (3)	0.4155 (3)	0.36924 (18)	0.0302 (6)	
H21	0.9485	0.4916	0.3211	0.036*	
C22	0.4876 (2)	0.7197 (2)	0.45106 (16)	0.0209 (4)	
H22A	0.3974	0.7174	0.4483	0.025*	
H22B	0.5011	0.7332	0.5132	0.025*	
C23	0.4757 (2)	0.8415 (2)	0.38565 (16)	0.0190 (4)	
C24	0.3880 (2)	0.8883 (2)	0.30771 (16)	0.0227 (5)	
H24	0.3403	0.8397	0.2948	0.027*	
C25	0.3701 (3)	1.0057 (3)	0.24878 (18)	0.0310 (6)	
H25	0.3087	1.0385	0.1964	0.037*	
C26	0.4414 (3)	1.0743 (3)	0.2664 (2)	0.0361 (6)	
H26	0.4290	1.1543	0.2258	0.043*	
C27	0.5305 (3)	1.0283 (3)	0.3421 (2)	0.0373 (7)	
H27	0.5805	1.0756	0.3531	0.045*	
C28	0.5472 (3)	0.9122 (3)	0.40263 (19)	0.0281 (5)	
H28	0.6073	0.8812	0.4555	0.034*	

### Atomic displacement parameters (Å^2^)

**Table:**

	*U*^11^	*U*^22^	*U*^33^	*U*^12^	*U*^13^	*U*^23^
Sn1	0.01704 (8)	0.01775 (8)	0.01609 (8)	−0.00884 (6)	0.00230 (5)	−0.00479 (6)
Cl1	0.0173 (2)	0.0304 (3)	0.0187 (2)	−0.0110 (2)	0.00222 (19)	−0.0022 (2)
Cl2	0.0230 (3)	0.0175 (2)	0.0230 (3)	−0.0069 (2)	0.0030 (2)	−0.0033 (2)
Cl3	0.0194 (3)	0.0212 (3)	0.0227 (3)	−0.0080 (2)	0.0060 (2)	−0.0054 (2)
Cl4	0.0408 (3)	0.0343 (3)	0.0217 (3)	−0.0275 (3)	0.0037 (2)	−0.0089 (2)
S1	0.0149 (2)	0.0185 (3)	0.0168 (2)	−0.0072 (2)	0.00010 (18)	−0.00387 (19)
S2	0.0157 (2)	0.0143 (2)	0.0170 (2)	−0.0053 (2)	0.00029 (18)	−0.00397 (19)
O1	0.0186 (8)	0.0201 (8)	0.0166 (7)	−0.0080 (6)	0.0013 (6)	−0.0057 (6)
O2	0.0187 (8)	0.0192 (8)	0.0167 (7)	−0.0091 (6)	0.0022 (6)	−0.0050 (6)
C1	0.0229 (11)	0.0199 (11)	0.0238 (11)	−0.0110 (9)	0.0006 (9)	−0.0024 (9)
C2	0.0210 (11)	0.0222 (11)	0.0277 (12)	−0.0127 (10)	0.0005 (9)	−0.0015 (9)
C3	0.0250 (12)	0.0276 (12)	0.0287 (12)	−0.0150 (10)	0.0045 (10)	−0.0053 (10)
C4	0.0252 (13)	0.0325 (14)	0.0443 (16)	−0.0150 (11)	0.0114 (11)	−0.0076 (12)
C5	0.0230 (13)	0.0426 (17)	0.0575 (19)	−0.0210 (13)	0.0022 (12)	−0.0066 (14)
C6	0.0311 (14)	0.0475 (17)	0.0430 (16)	−0.0254 (13)	−0.0047 (12)	−0.0085 (13)
C7	0.0284 (13)	0.0331 (14)	0.0331 (13)	−0.0188 (11)	0.0038 (10)	−0.0094 (11)
C8	0.0152 (10)	0.0269 (12)	0.0244 (11)	−0.0095 (9)	−0.0021 (8)	−0.0075 (9)
C9	0.0120 (10)	0.0251 (12)	0.0250 (11)	−0.0072 (9)	−0.0019 (8)	−0.0059 (9)
C10	0.0175 (11)	0.0263 (12)	0.0287 (12)	−0.0086 (10)	0.0006 (9)	−0.0039 (10)
C11	0.0231 (12)	0.0369 (15)	0.0272 (13)	−0.0104 (11)	0.0055 (10)	−0.0059 (11)
C12	0.0240 (13)	0.0346 (15)	0.0388 (15)	−0.0092 (11)	0.0083 (11)	−0.0165 (12)
C13	0.0202 (12)	0.0240 (12)	0.0442 (15)	−0.0075 (10)	0.0032 (10)	−0.0082 (11)
C14	0.0186 (11)	0.0268 (12)	0.0288 (12)	−0.0088 (10)	−0.0003 (9)	−0.0024 (10)
C15	0.0179 (10)	0.0185 (11)	0.0246 (11)	−0.0067 (9)	−0.0013 (8)	−0.0066 (9)
C16	0.0156 (10)	0.0241 (12)	0.0268 (12)	−0.0072 (9)	−0.0015 (9)	−0.0094 (9)
C17	0.0222 (12)	0.0270 (13)	0.0326 (13)	−0.0072 (10)	0.0005 (10)	−0.0070 (10)
C18	0.0272 (14)	0.0254 (13)	0.0441 (16)	−0.0041 (11)	−0.0076 (12)	−0.0028 (12)
C19	0.0205 (13)	0.0331 (15)	0.0479 (17)	0.0003 (11)	−0.0049 (11)	−0.0187 (13)
C20	0.0239 (13)	0.0480 (18)	0.0355 (15)	−0.0049 (13)	0.0027 (11)	−0.0174 (13)
C21	0.0240 (12)	0.0341 (14)	0.0273 (13)	−0.0087 (11)	−0.0005 (10)	−0.0082 (11)
C22	0.0191 (11)	0.0151 (10)	0.0226 (11)	−0.0032 (9)	0.0040 (8)	−0.0051 (9)
C23	0.0173 (10)	0.0158 (10)	0.0231 (11)	−0.0062 (8)	0.0042 (8)	−0.0081 (8)
C24	0.0207 (11)	0.0186 (11)	0.0263 (12)	−0.0062 (9)	0.0010 (9)	−0.0078 (9)
C25	0.0342 (14)	0.0223 (12)	0.0239 (12)	−0.0040 (11)	0.0007 (10)	−0.0022 (10)
C26	0.0357 (15)	0.0210 (12)	0.0437 (16)	−0.0098 (11)	0.0121 (12)	0.0007 (11)
C27	0.0305 (14)	0.0231 (13)	0.065 (2)	−0.0169 (11)	0.0101 (13)	−0.0136 (13)
C28	0.0211 (12)	0.0222 (12)	0.0411 (15)	−0.0083 (10)	−0.0038 (10)	−0.0111 (11)

### Geometric parameters (Å, °)

**Table:**

Sn1—O2	2.0938 (16)	C11—H11	0.9500
Sn1—O1	2.1176 (16)	C12—C13	1.383 (4)
Sn1—Cl3	2.3772 (5)	C12—H12	0.9500
Sn1—Cl2	2.3800 (6)	C13—C14	1.389 (4)
Sn1—Cl4	2.4045 (6)	C13—H13	0.9500
Sn1—Cl1	2.4297 (5)	C14—H14	0.9500
S1—O1	1.5502 (16)	C15—C16	1.508 (3)
S1—C1	1.809 (2)	C15—H15A	0.9900
S1—C8	1.822 (2)	C15—H15B	0.9900
S2—O2	1.5558 (16)	C16—C21	1.386 (4)
S2—C15	1.810 (2)	C16—C17	1.387 (4)
S2—C22	1.816 (2)	C17—C18	1.388 (4)
C1—C2	1.503 (3)	C17—H17	0.9500
C1—H1A	0.9900	C18—C19	1.382 (4)
C1—H1B	0.9900	C18—H18	0.9500
C2—C7	1.387 (4)	C19—C20	1.375 (5)
C2—C3	1.400 (3)	C19—H19	0.9500
C3—C4	1.379 (4)	C20—C21	1.393 (4)
C3—H3	0.9500	C20—H20	0.9500
C4—C5	1.383 (4)	C21—H21	0.9500
C4—H4	0.9500	C22—C23	1.495 (3)
C5—C6	1.383 (4)	C22—H22A	0.9900
C5—H5	0.9500	C22—H22B	0.9900
C6—C7	1.388 (4)	C23—C28	1.393 (3)
C6—H6	0.9500	C23—C24	1.394 (3)
C7—H7	0.9500	C24—C25	1.390 (4)
C8—C9	1.498 (3)	C24—H24	0.9500
C8—H8A	0.9900	C25—C26	1.374 (4)
C8—H8B	0.9900	C25—H25	0.9500
C9—C10	1.389 (3)	C26—C27	1.377 (5)
C9—C14	1.397 (3)	C26—H26	0.9500
C10—C11	1.386 (4)	C27—C28	1.394 (4)
C10—H10	0.9500	C27—H27	0.9500
C11—C12	1.386 (4)	C28—H28	0.9500
			
O2—Sn1—O1	81.00 (6)	C10—C11—H11	119.8
O2—Sn1—Cl3	88.98 (4)	C12—C11—H11	119.8
O1—Sn1—Cl3	88.48 (4)	C13—C12—C11	119.5 (3)
O2—Sn1—Cl2	92.70 (4)	C13—C12—H12	120.3
O1—Sn1—Cl2	173.13 (4)	C11—C12—H12	120.3
Cl3—Sn1—Cl2	94.17 (2)	C12—C13—C14	120.5 (3)
O2—Sn1—Cl4	169.67 (5)	C12—C13—H13	119.7
O1—Sn1—Cl4	88.95 (4)	C14—C13—H13	119.7
Cl3—Sn1—Cl4	93.16 (2)	C13—C14—C9	120.2 (2)
Cl2—Sn1—Cl4	97.22 (2)	C13—C14—H14	119.9
O2—Sn1—Cl1	86.64 (4)	C9—C14—H14	119.9
O1—Sn1—Cl1	85.29 (4)	C16—C15—S2	107.39 (16)
Cl3—Sn1—Cl1	172.87 (2)	C16—C15—H15A	110.2
Cl2—Sn1—Cl1	91.656 (19)	S2—C15—H15A	110.2
Cl4—Sn1—Cl1	90.18 (2)	C16—C15—H15B	110.2
O1—S1—C1	101.73 (10)	S2—C15—H15B	110.2
O1—S1—C8	103.26 (10)	H15A—C15—H15B	108.5
C1—S1—C8	101.23 (11)	C21—C16—C17	119.5 (2)
O2—S2—C15	100.62 (10)	C21—C16—C15	120.5 (2)
O2—S2—C22	104.75 (10)	C17—C16—C15	119.9 (2)
C15—S2—C22	101.73 (11)	C16—C17—C18	120.1 (3)
S1—O1—Sn1	121.84 (9)	C16—C17—H17	119.9
S2—O2—Sn1	121.81 (9)	C18—C17—H17	119.9
C2—C1—S1	108.98 (16)	C19—C18—C17	120.2 (3)
C2—C1—H1A	109.9	C19—C18—H18	119.9
S1—C1—H1A	109.9	C17—C18—H18	119.9
C2—C1—H1B	109.9	C20—C19—C18	119.9 (3)
S1—C1—H1B	109.9	C20—C19—H19	120.1
H1A—C1—H1B	108.3	C18—C19—H19	120.1
C7—C2—C3	119.4 (2)	C19—C20—C21	120.3 (3)
C7—C2—C1	120.7 (2)	C19—C20—H20	119.8
C3—C2—C1	119.8 (2)	C21—C20—H20	119.8
C4—C3—C2	120.3 (2)	C16—C21—C20	119.9 (3)
C4—C3—H3	119.9	C16—C21—H21	120.0
C2—C3—H3	119.9	C20—C21—H21	120.0
C3—C4—C5	120.0 (3)	C23—C22—S2	114.98 (16)
C3—C4—H4	120.0	C23—C22—H22A	108.5
C5—C4—H4	120.0	S2—C22—H22A	108.5
C4—C5—C6	120.2 (3)	C23—C22—H22B	108.5
C4—C5—H5	119.9	S2—C22—H22B	108.5
C6—C5—H5	119.9	H22A—C22—H22B	107.5
C5—C6—C7	120.2 (3)	C28—C23—C24	119.2 (2)
C5—C6—H6	119.9	C28—C23—C22	121.2 (2)
C7—C6—H6	119.9	C24—C23—C22	119.6 (2)
C6—C7—C2	120.0 (3)	C25—C24—C23	120.2 (2)
C6—C7—H7	120.0	C25—C24—H24	119.9
C2—C7—H7	120.0	C23—C24—H24	119.9
C9—C8—S1	113.76 (16)	C26—C25—C24	119.9 (3)
C9—C8—H8A	108.8	C26—C25—H25	120.1
S1—C8—H8A	108.8	C24—C25—H25	120.1
C9—C8—H8B	108.8	C25—C26—C27	120.8 (3)
S1—C8—H8B	108.8	C25—C26—H26	119.6
H8A—C8—H8B	107.7	C27—C26—H26	119.6
C10—C9—C14	118.9 (2)	C26—C27—C28	119.8 (3)
C10—C9—C8	120.2 (2)	C26—C27—H27	120.1
C14—C9—C8	120.9 (2)	C28—C27—H27	120.1
C11—C10—C9	120.6 (2)	C23—C28—C27	120.0 (2)
C11—C10—H10	119.7	C23—C28—H28	120.0
C9—C10—H10	119.7	C27—C28—H28	120.0
C10—C11—C12	120.3 (2)		
			
C1—S1—O1—Sn1	−136.20 (11)	C9—C10—C11—C12	0.3 (4)
C8—S1—O1—Sn1	119.12 (11)	C10—C11—C12—C13	−0.1 (4)
O2—Sn1—O1—S1	140.81 (11)	C11—C12—C13—C14	−0.2 (4)
Cl3—Sn1—O1—S1	51.60 (10)	C12—C13—C14—C9	0.2 (4)
Cl4—Sn1—O1—S1	−41.59 (10)	C10—C9—C14—C13	0.1 (4)
Cl1—Sn1—O1—S1	−131.86 (10)	C8—C9—C14—C13	179.4 (2)
C15—S2—O2—Sn1	138.94 (11)	O2—S2—C15—C16	−73.55 (17)
C22—S2—O2—Sn1	−115.80 (11)	C22—S2—C15—C16	178.78 (16)
O1—Sn1—O2—S2	132.52 (11)	S2—C15—C16—C21	92.2 (2)
Cl3—Sn1—O2—S2	−138.85 (10)	S2—C15—C16—C17	−84.7 (2)
Cl2—Sn1—O2—S2	−44.73 (10)	C21—C16—C17—C18	0.1 (4)
Cl4—Sn1—O2—S2	119.0 (2)	C15—C16—C17—C18	177.0 (2)
Cl1—Sn1—O2—S2	46.77 (10)	C16—C17—C18—C19	0.4 (4)
O1—S1—C1—C2	77.36 (18)	C17—C18—C19—C20	−0.6 (4)
C8—S1—C1—C2	−176.37 (17)	C18—C19—C20—C21	0.3 (5)
S1—C1—C2—C7	−96.3 (2)	C17—C16—C21—C20	−0.4 (4)
S1—C1—C2—C3	80.0 (2)	C15—C16—C21—C20	−177.3 (2)
C7—C2—C3—C4	0.1 (4)	C19—C20—C21—C16	0.2 (4)
C1—C2—C3—C4	−176.3 (2)	O2—S2—C22—C23	−35.0 (2)
C2—C3—C4—C5	−0.3 (4)	C15—S2—C22—C23	69.5 (2)
C3—C4—C5—C6	−0.1 (4)	S2—C22—C23—C28	−90.2 (2)
C4—C5—C6—C7	0.8 (5)	S2—C22—C23—C24	92.0 (2)
C5—C6—C7—C2	−1.1 (4)	C28—C23—C24—C25	−1.3 (4)
C3—C2—C7—C6	0.6 (4)	C22—C23—C24—C25	176.6 (2)
C1—C2—C7—C6	177.0 (2)	C23—C24—C25—C26	1.4 (4)
O1—S1—C8—C9	41.3 (2)	C24—C25—C26—C27	−0.2 (4)
C1—S1—C8—C9	−63.7 (2)	C25—C26—C27—C28	−1.1 (4)
S1—C8—C9—C10	−86.7 (2)	C24—C23—C28—C27	0.1 (4)
S1—C8—C9—C14	94.0 (2)	C22—C23—C28—C27	−177.8 (2)
C14—C9—C10—C11	−0.3 (4)	C26—C27—C28—C23	1.1 (4)
C8—C9—C10—C11	−179.6 (2)		
